# Stress Odorant Sensory Response Dysfunction in *Drosophila* Fragile X Syndrome Mutants

**DOI:** 10.3389/fnmol.2018.00242

**Published:** 2018-08-08

**Authors:** Alaura Androschuk, Richard X. He, Savannah Weber, Cory Rosenfelt, Francois V. Bolduc

**Affiliations:** ^1^Department of Pediatrics, University of Alberta, Edmonton, AB, Canada; ^2^Neuroscience and Mental Health Institute, University of Alberta, Edmonton, AB, Canada; ^3^Department of Medical Genetics, University of Alberta, Edmonton, AB, Canada

**Keywords:** Fragile X syndrome, sensory response dysfunction, *Drosophila*, cAMP, cGMP, avoidance response, IBMX

## Abstract

Sensory processing dysfunction (SPD) is present in most patients with intellectual disability (ID) and autism spectrum disorder (ASD). Silencing expression of the Fragile X mental retardation 1 (*FMR1*) gene leads to Fragile X syndrome (FXS), the most common single gene cause of ID and ASD. *Drosophila* have a highly conserved FMR1 ortholog, *dfmr1*. *dfmr1* mutants display cognitive and social defects reminiscent of symptoms seen in individuals with FXS. We utilized a robust behavioral assay for sensory processing of the *Drosophila* stress odorant (dSO) to gain a better understanding of the molecular basis of SPD in FXS. Here, we show that *dfmr1* mutant flies present significant defects in dSO response. We found that *dfmr1* expression in mushroom bodies is required for dSO processing. We also show that cyclic adenosine monophosphate (cAMP) signaling via PKA is activated after exposure to dSO and that several drugs regulating both cAMP and cyclic guanosine monophosphate (cGMP) levels significantly improved defects in dSO processing in *dfmr1* mutant flies.

## Introduction

Sensory processing dysfunction (SPD) is a key symptom seen in 90% of individuals with intellectual disability (ID) and autism spectrum disorder (ASD) (Marco et al., 2011; Chang et al., 2014) where the response to a given stimulus is different from the typically developing population. The most common clinical features of SPD are under-responsiveness, sensory seeking, auditory filtering, and tactile sensitivity (Tomchek and Dunn, 2007). This reflects that multiple senses are affected, including audition, touch, vision, and oral sensation (Kern et al., 2006). For instance, some individuals with ASD will perceive sound much louder than typically developing individuals and this will affect their behavior. Indeed, they will either block their ears or become increasingly anxious. SPD affects patients with mild to severe ID equally (Engel-Yeger et al., 2011). Sensory processing deficits have also been linked to stereotypical movements and anxiety (Joosten and Bundy, 2010). SPD predicts communication competence and maladaptive behaviors (Lane et al., 2010), which are the drivers of socio-economic impact (Bailey et al., 2012). Importantly, SPD is present in both children and adults (Crane et al., 2009). While brain magnetic resonance imaging (MRI) studies have provided some insights (Owen et al., 2013), the molecular basis and treatment of SPD remain poorly understood.

Fragile X syndrome (FXS) is the most common single gene cause of ID and ASD (Androschuk et al., 2015). FXS is caused by a trinucleotide CGG repeat expansion that leads to the methylation and transcriptional silencing of the Fragile X mental retardation 1 (*FMR1*) gene. This results in the loss of Fragile X mental retardation protein (FMRP), an mRNA-binding protein that functions in neuronal mRNA metabolism, namely in the translation of neuronal mRNAs involved in synaptic structure and function. Individuals with FXS frequently present with SPD (Goldson, 2001), which has a major impact on their ability to function (Baranek et al., 2002). We reasoned that response to sensory stimulation may serve as endophenotype of the processing of information.

*Drosophila* have a conserved *FMR1* ortholog, *dfmr1*. *dfmr1* mutants present with the circadian, cognitive, and social defects also observed in individuals with FXS. Little is known about the response to sensory signal in *dfmr1* mutant flies. Normal shock and olfactory stimuli used for olfactory memory training have not provided a model to study sensory processing. Suh et al. (2004) discovered that *Drosophila* avoided systematically an environment in which other flies had previously been submitted to mechanical stress. Indeed, the *Drosophila* stress odorant (dSO) is a signal emitted when flies are subjected to electrical or mechanical stressors, and elicits an innate and robust avoidance behavioral response in wild-type (WT) *Drosophila* (Suh et al., 2004). Here, we show that *Drosophila dfmr1* mutant flies present significant defect in responding to dSO.

## Materials and Methods

### *Drosophila melanogaster* Stocks and Crosses

Fly stocks were maintained at 22°C on standard cornmeal-yeast media from Cold Spring Harbor Laboratory. WT stocks were backcrossed to *w*^1118^*isoCJ1* for 6 generations. *dfmr1*^B55^ flies were obtained from Dr. Kendal Broadie (Vanderbilt University). *dfmr1*^3^ flies and *dfmr1*^3^ flies containing a WT rescue transgene (*dfmr1*^3^*WTR*) were obtained from Dr. Tom Jongens (University of Pennsylvania). *Elav-Gal4, OK107-Gal4, C747-Gal4, MB247-Gal4*, and *Feb170-Gal4* flies were obtained from Dr. Tim Tully. To determine the spatial requirement of FMRP in mediating dSO avoidance, we used RNA interference (RNAi) against FMRP in order to knockdown/reduce expression of FMRP. Using the Gal4-UAS system (Brand and Perrimon, 1993), we generated crosses by mating *Elav(Embryonic lethal vision)-Gal4, OK107-Gal4, Feb170-Gal4, MB247-Gal4, and 747-Gal4* virgin females to *UAS-dfmr1RNAi*^1-7^ males generated previously in our laboratory (Bolduc et al., 2008). To assess the spatio-temporal requirement of *dfmr1*, we used Gal80^ts^; Elav-Gal4 (from Dr. Tom Jongens) to drive the expression *UAS-dfmr1RNAi*^1-7^. WT and transgenic flies were tested in parallel. WT and transgenic flies were raised at 18°C (restricting the expression of ELAV-Gal4) and then transferred for 3 days at 30°C allowing its expression, or kept at 18°C, to restrict the expression of ELAV-Gal4, as before for memory experiments in our laboratory (Bolduc et al., 2008).

### Behavioral Paradigm

The T maze avoidance assay was conducted, as previously described by Suh et al. (2004), with modifications (Androschuk, 2016). All testing was performed in an environment controlled room which was maintained at 25°C and 70% humidity. To produce dSO a group of 50 flies (mixed sex, termed ‘emitter’ flies) were vortexed (Fisher Vortex Mixer) for 1 min (alternating between 3 s of vortexing followed by 5 s of rest for the entire duration) in a 10 mL Falcon tube sealed with Parafilm (Fisher Scientific 149598) at maximum speed. Emitter flies were then removed from the Falcon tube and the dSO-containing Falcon tube was placed into a T maze. A new dSO-free Falcon tube was placed opposite the dSO-containing tube. Subsequently, 50 naïve flies (termed ‘responder’) were transferred into a new Falcon tube and loaded into the elevator of the T maze. Responder flies were then given 1 min to choose between the dSO-containing and the dSO-free Falcon tubes. Following the 1-min testing period, flies were sequestered and avoidance response was scored. Avoidance was scored as a Performance Index (PI), calculated as follows:

$$\text{PI}=\frac{\left(\text{No. of responder flies in dSO-free tube})-(\text{No. of responder flies in dSO tube}\right)}{\text{Total no. of responder flies}}$$

### Statistical Analysis

For unplanned comparisons between more than 2 groups, we used one-way ANOVA followed by Tukey’s test. For all analysis between 2 groups, we used a two-tailed Student’s *t*-test. Analysis was performed using GraphPad (PRISM7).

### CO_2_ Avoidance

Gaseous CO_2_ was used in place of emitter flies in CO_2_ avoidance testing. A flow-meter set at 0.2 mL/min or 0.5 mL/min was used to administer CO_2_ into Falcon tubes for 30 s, which were then momentarily sealed using Parafilm prior to being loaded into the T maze. Responder flies were given 1 min to choose between the CO_2_-containing and the CO_2_-free Falcon tubes. Flies were then sequestered and avoidance response was scored as a PI, as above.

### Drug Administration

Using previously published feeding protocols for Lithium in the classical olfactory conditioning assay (Choi et al., 2015), we performed dose response curves for the avoidance assay. For drugs not previously tested in our laboratory (IBMX, dipyridamole, 8-CPT), we assessed response at 1 day as well as longer treatment if there was no response after 1 day. The treatments were provided only in post-natal set up to reflect the potential clinical application at this time. For all experiments, only responder flies were treated with vehicle or treatment.

#### IBMX

The 3-isobutyl-1-methylxanthine (IBMX; Sigma I7018) was added to standard food media for drug administration. The 1-day-old flies were placed in food bottles containing 0.05 mg/mL IBMX or the food alone for 4 days and transferred to food vials containing 0.05 mg/mL IMBX or the food alone the day prior to testing (Androschuk, 2016).

#### 8-CPT

The 8-(4-Chlorophenylthio)adenosine 3′,5′-cyclic monophosphate sodium salt (8-CPT; Sigma C3912) was administered to flies on 2.1-cm Whatman filter paper (Fisher WHT1540321). The 1-day-old flies were placed in vials containing 225 μL of 8-CPT with 5% sucrose or 5% sucrose only and treated for 5 days prior to testing. Flies were transferred daily to new vials containing fresh 8-CPT with sucrose or sucrose alone (Androschuk, 2016).

#### LiCl

Lithium chloride (LiCl; Sigma L9650) was added directly to the standard food media for drug administration. The 1-day-old flies were set up in food bottles containing 10 mM LiCl or the food alone for 4 days and transferred to food vials containing 10 mM LiCl or the food alone the day prior to testing (Androschuk, 2016).

#### Dipyridamole

The 0.8 mM dipyridamole (Sigma D9766) was added directly to standard food media for drug administration with 0.8% DMSO. The 1-day-old flies were placed for 24 h in food bottles containing either dipyridamole or vehicle.

### Immunohistochemistry

After 1 min exposure to dSO, naïve responder flies avoiding the dSO were placed on ice for 2 min and heads of female responder flies were removed and placed in cold PBS for dissection. Unexposed flies were placed in a dSO-free 10 mL Falcon tube sealed with Parafilm for 1 min. Then flies were processed blind in parallel. Flies were then placed on ice for 2 min and heads of female flies were removed and placed in cold PBS for dissection. Fly heads were dissected as before (Bolduc et al., 2008). Protein kinase A (PKA) was identified with 1:1000 α-PKA catalytic subunit (phospho T198) (Abcam ab118531).

Following overnight incubation with the secondary antibody (1:200 Cy3 α-Rabbit Jackson ImmunoResearch 111-165-003) and 1% PBS triton (PBST) with 0.25% NGS, brains were washed three times with 1% PBST and mounted using FocusClear (Cedarlane FC-101). Imaging was completed using a Zeiss LSM 700 Confocal Microscope and images were quantified using ImageJ (Androschuk, 2016). Gain was set the same for both groups.

### Pathway Analysis

*In silico* pathway analyses were performed with Ingenuity Pathway Analysis (IPA, Qiagen) to identify interactions with cAMP and cGMP by genes associated with ASD from the SFARI Gene database (https://www.sfari.org/) and genes implicated in ID from published literature (Gilissen et al., 2014).

## Results

### *dfmr1* Is Required for dSO Response

**FIGURE 1:**
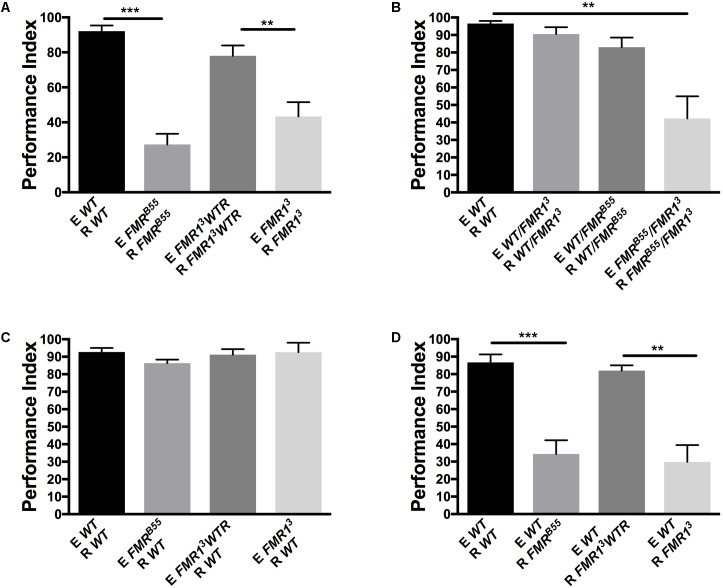
Fragile X mental retardation protein (FMRP) is required for avoidance of *Drosophila melanogaster* stress odorant (dSO). For all figures, the flies emitting the dSO (E) are submitted to the vortexing protocol. The flies tested for their response to tubes exposed to dSO or not are considered the responders (R) of dSO signaling. **(A)** *FMR*^B55^ mutants exhibit a significantly lower avoidance in response to dSO compared to WT flies (Student’s *t*-test *P* < 0.0001; *N* = 8); avoidance is quantified as Performance Index (PI). *FMR1*^3^ exhibit decreased avoidance compared to *FMR1*^3^*WTR* flies, the avoidance of which is rescued genetically through the addition of a genomic *dfmr1*^3^ fragment (Student’s *t*-test *P* = 0.0049; *N* = 8). dSO avoidance behavior is scored as PI. **(B)** *FMR*^B55^*/FMR1*^3^ flies exhibit decreased avoidance compared to WT flies (Tukey’s test *P* = 0.0001; *N* = 7). Avoidance behavior is genetically rescued in *FMR*^B55^*/WT* (Tukey *P* = 0.9348; *N* = 7) and *FMR1*^3^*/WT* (ANOVA *P* = 0.5638; *N* = 7) flies. *FMR*^B55^*/FMR1*^3^ flies exhibit decrease avoidance behavior compared to *FMR1*^3^*/WT (*Tukey’s test *P* = 0.0004; *N* = 7) and *FMR*^B55^*/WT* (Tukey’s test *P* = 0.0028; *N* = 7) flies. **(C)** WT flies did not exhibit decreased avoidance behavior to dSO emitted by *FMR*^B55^, (Student’s *t*-test *P* = 0.0988; *N* = 5), *FMR1*^3^ (Student’s *t*-test *P* = 0.9897; *N* = 5), and *FMR1*^3^*WTR* flies (Student’s *t*-test *P* = 0.7153; *N* = 5). **(D)** *FMR*^B55^ flies exhibit decreased avoidance behavior to WT dSO (Student’s *t*-test *P* < 0.0001; *N* = 12). *FMR1*^3^ also flies exhibit diminished avoidance behavior to WT dSO as compared to *FMR1*^3^*WTR* flies (Student’s *t*-test *P* = 0.0018; *N* = 12). ^∗∗^*P* < 0.01, ^∗∗∗^*P* < 0.001.

In order to determine the role of FMRP in the processing and modulation of dSO avoidance behavior in *Drosophila*, we utilized the two null alleles, *dfmr1*^3^ and *dfmr1*^B55^, known to have olfactory and courtship memory defects, as well as social interaction defect (McBride et al., 2005; Bolduc et al., 2008; Bolduc et al., 2010). We found that *dfmr1*^3^ and *dfmr1*^B55^ flies exhibited a significant decrease in dSO avoidance compared to flies with the appropriate genetic control (*dfrm1*^3^ with a genomic rescue fragment, *FMR1*^3^*WTR*, and WT flies) (**Figure 1A**). Similarly, transheterozygous *FMR*^B55^*/FMR1*^3^ mutants exhibited a significant decrease in dSO avoidance behavior compared to WT flies (**Figure 1B**). Next, we tested if FMRP was involved in dSO emission or dSO response. We conducted avoidance trials in which WT flies were utilized as the emitter or responder and tested with the *dfmr1* mutant flies. WT flies exhibited normal avoidance in response to dSO emitted by *FMR*^B55^ and *FMR1*^3^ (**Figure 1C**). *FMR*^B55^ and *FMR1*^3^ flies exhibited decreased avoidance as compared to their genetic controls when WT flies were utilized as emitter flies (**Figure 1D**). Considering the normal avoidance of WT flies when using *dfmr1* flies as emitters, we considered that FMRP is involved in sensory processing and not emission of dSO.

### *dfmr1* Is Required in Mushroom Bodies (MB) for dSO Processing

**FIGURE 2:**
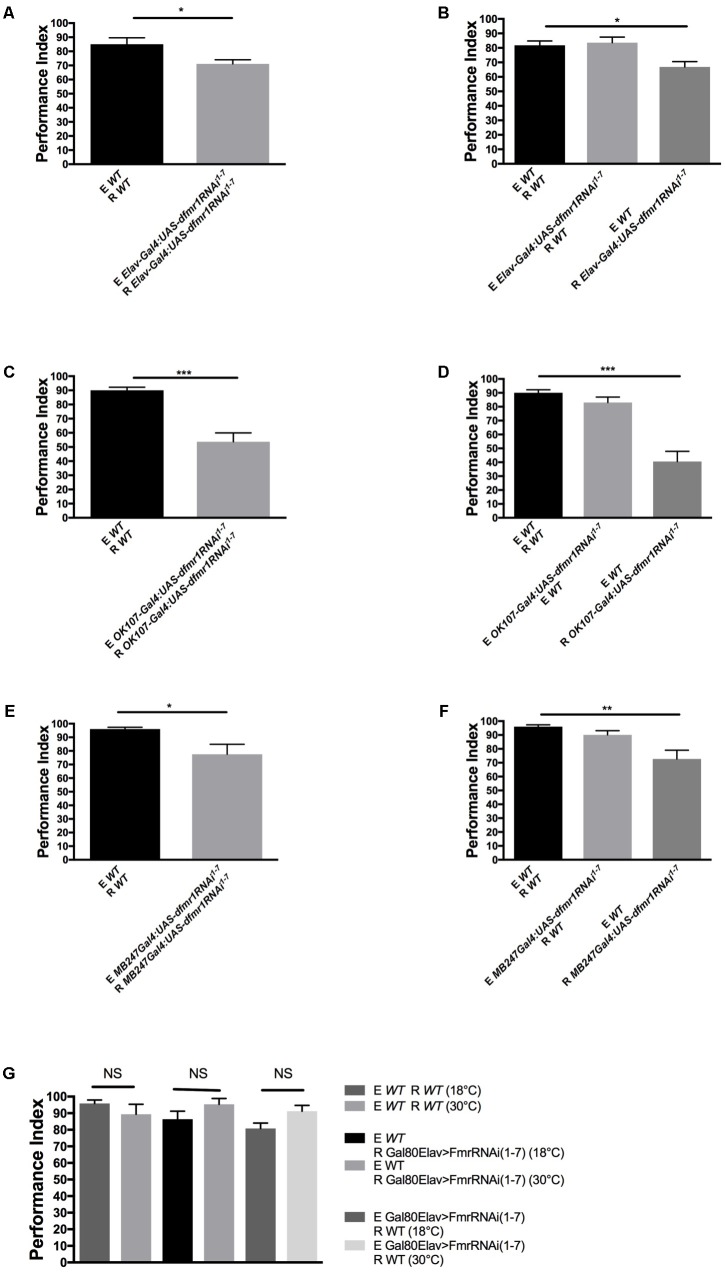
FMRP expression in mushroom bodies and glia is required for dSO avoidance. **(A)** Pan-neuronal knockdown of FMRP by *Elav-Gal4:UAS-dfmr1RNAi*^1-7^, results in decreased avoidance to dSO compared to WT flies (Student’s *t*-test *P* = 0.0409; *N* = 20). **(B)** WT flies did not exhibit any significant decrease in avoidance (*behavior to dSO emitted by *Elav-Gal4:UAS-dfmr1RNAi*^1-7^ flies (Student’s *t*-test *P* = 0.7653; *N* = 10). *Elav-Gal4:UAS-dfmr1RNAi*^1-7^ flies exhibit decreased avoidance behavior to WT dSO as compared to WT flies (Student’s *t*-test *P* = 0.00285; *N* = 12). **(C)***OK107-Gal4:UAS-dfmr1RNAi*^1-7^ flies exhibit significantly decreased avoidance to dSO compared to WT flies (Student’s *t*-test *P* < 0.0001; *N* = 12). **(D)***OK107-Gal4:UAS-dfmr1RNAi*^1-7^ flies exhibit a significantly decreased avoidance response when tested against dSO emitted by WT flies (Student’s *t*-test *P* < 0.0001; *N* = 8). WT flies exhibited normal avoidance behavior when tested against dSO emitted by *OK107 > FmrRNAi*(1-7) flies (Student’s *t*-test *P* = 0.1240; *N* = 8). **(E)***MB247Gal4;UAS-dfmr1RNAi*^1-7^ flies exhibited diminished avoidance behavior as compared to WT flies (Student’s *t*-test *P* = 0.0239; *N* = 10) when tested with same genotype pairs. **(F)***MB247Gal4;UAS-dfmr1RNAi*^1-7^ flies exhibit a significantly decreased avoidance response when tested against dSO emitted by WT flies (Student’s *t*-test *P* = 0.0016; *N* = 8). WT flies exhibited normal avoidance behavior when tested against dSO emitted by *MB247Gal4;UAS-dfmr1RNAi*^1-7^ flies (Student’s *t*-test *P* = 0.0707; *N* = 8). **(G)** WT (Student’s *t*-test *P* = 0.27; *N* = 5) and *Gal80ts;ELAV-Gal4 > UAS-dfmr1RNAi*^1-7^ flies present no significant defect in avoidance performance comparing their performance at restrictive (18°C) versus permissive (30°C) either as responder to dSO (R) (Student’s *t*-test *P* = 0.1689; *N* = 5 PI per group) or as emitter of dSO (E) (Student’s *t*-test *P* = 0.059; *N* = 5 PI per group). NS, not significant. ^∗^*P* < 0.05, ^∗∗^*P* < 0.01*, ^∗∗∗^*P* < 0.001.

We first used the pan-neuronal driver *ELAV-Gal4* and *UAS-FMR* responder with RNAi to knockdown FMRP in neurons. Pan-neuronal knockdown of FMRP resulted in a significant decrease in dSO avoidance response, which we confirmed causes a dSO processing defect and not emission deficiency from knockdown of FMRP (**Figures 2A,B**). Next, we asked whether loss of FMRP in two higher-order processing centers, the mushroom bodies (MB) and the central complex, are involved in dSO avoidance. We showed previously that FMRP was required in MB for olfactory memory (Bolduc et al., 2008). Bräcker et al. (2013) showed that MB were required for CO_2_ avoidance response in the context of food deprivation or food-related odors. Knockdown of FMRP using the MB-specific driver OK107 resulted in a significantly decreased avoidance response compared to WT flies (**Figures 2C,D**). To confirm the requirement of FMRP in the MB in mediating dSO avoidance behavior, we utilized the MB-specific driver MB247 to knockdown FMRP, which resulted in a significant defect in dSO avoidance (**Figures 2E,F**). Unlike the significant decrease in dSO avoidance that resulted from using the *OK107-Gal4* and *MB247-Gal4* driver lines to knockdown FMRP in the MB, use of the C*747-Gal4* driver line did not result in a significant decrease in dSO avoidance (results not shown). These differences are likely due to regional specificity and strength of expression of each individual driver within the MB. The *OK107-Gal4* and *MB247-Gal4* driver lines strongly target expression in α, β, and γ Kenyon cells, while *C747-Gal4* expression is weaker in γ Kenyon cells (Aso et al., 2009). Knockdown of FMRP in the central complex using *FEB170-Gal4* did not result in any significant changes in dSO avoidance (**Supplementary Figures 1A,B**). In addition, we did not observe significant defects in the avoidance after post-natal variation in FMRP levels [using Gal80^ts^; ELAV-gal4 with *UAS-dfmr1RNAi*^1-7^ to lower FMRP level as before (Bolduc et al., 2008)], which is different to what was observed in long-term olfactory memory defects in *dfmr1* mutants previously and more similar to short-term memory (**Figure 2G**; Bolduc et al., 2008).

### Targeting cAMP/cGMP Signaling Pharmacologically in Adult Flies Rescues dSO Response in *dfmr1* Mutants

Next, we explored if pharmacological intervention could improve *dfmr1* mutant avoidance response and help decipher the molecular mechanism related to the dSO defects in *dfmr1* mutant flies. We first considered the seminal report from Suh et al. (2004) who showed that CO_2_ was a key component of the dSO. Lin et al. (2013) further showed that CO_2_ olfactory information was conveyed by 2 types of projection neurons depending on the concentration of CO_2_ present in the environment (Lin et al., 2013). We therefore tested response to CO_2_ for *dfmr1* mutants and found that *dfmr1*^3^ and *dfmr1*^B55^ had significant response deficits to CO_2_ at 0.2 mL/min and 0.5 mL/min (**Supplementary Figures 1C,D**). As cAMP signaling is required for CO_2_ sensing (Klengel et al., 2005) and cAMP signaling dysregulation is linked to FXS early on in human (Berry-Kravis et al., 1984) and in *Drosophila* (Kanellopoulos et al., 2012), we investigated if cAMP regulation could be involved in the defective dSO response in *dfmr1* mutants. Activity dependent reactivity of cAMP is abnormal in FXS (Berry-Kravis et al., 1995). Moreover, FMRP binds to adenylyl cyclase (AC) and phosphodiesterase (PDE) mRNAs (Darnell et al., 2011). Importantly, PDE4 inhibitors Rolipram and Lithium, which lead to increased cAMP levels, have been found to rescue memory and long-term depression (LTD) defects in FXS mice and flies (Choi et al., 2015, 2016).

**FIGURE 3:**
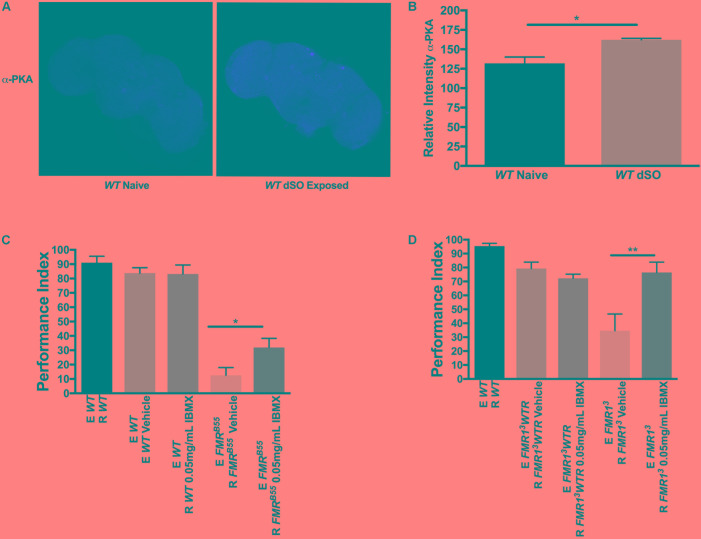
Pharmacological intervention targeting cyclic adenosine monophosphate (cAMP) rescues dSO avoidance in Fragile X syndrome flies. **(A)** Confocal imaging of WT flies catalytic subunit PKA (phospho T198) levels in dSO exposed and unexposed WT fly brains processed in parallel and imaged with same gain. **(B)** dSO exposure results in an overall significant increase in PKA catalytic subunit (phospho T198) levels in WT brains compared to unexposed control (Student’s *t*-test *P* = 0.0226; *N* = 3). All graphs depict mean ± SEM. **(C)** 5-day treatment of *FMR*^B55^ flies with 0.05 mg/mL IBMX results in significantly increased avoidance compared to *FMR*^B55^ on vehicle (Student’s *t*-test *P* = 0.0282; *N* = 14). No significant difference in avoidance behavior observed in WT flies following 5-day treatment with 0.05 mg/mL IBMX as compared to vehicle (Student’s *t*-test *P* = 0.9379; *N* = 14). **(D)** 5-day treatment of *FMR1*^3^ flies with 0.05 mg/mL IBMX resulted in a significantly increase in avoidance compared to *FMR1*^3^ fed vehicle (Student’s *t*-test *P* = 0.0068; *N* = 13). No significant difference in avoidance behavior observed in *FMR1*^3^*WTR* flies following 5-day treatment with 0.05 mg/mL IBMX as compared to vehicle (Student’s *t*-test *P* = 0.02077; *N* = 13). ^∗^*P* < 0.05, ^∗∗^*P* < 0.01.

We assessed if dSO exposure was associated with activation of the cAMP pathway using brain immunohistochemistry first. Activation of cAMP leads to concomitant activation of cAMP-dependent protein kinase A (PKA). Using confocal imaging, we examined the relative levels of the phosphorylated catalytic subunit of PKA in WT fly brains in response to dSO exposure by utilizing a free catalytic subunit-specific PKA (phospho T198) antibody. PKA catalytic subunit mRNA and protein have been shown to be expressed throughout the brain with increased signal in the MB Kenyon cells especially in the dorsal aspect (Skoulakis et al., 1993). PKA is activated when cAMP binds to regulatory subunits, resulting in the disassociation of catalytic subunits. The catalytic-PKA phosphorylation levels were significantly elevated overall in WT brains following dSO exposure compared to naïve, unexposed WT flies, suggesting that cAMP signaling participates in modulating dSO avoidance behavior (**Figures 3A,B**). Interestingly, high expression was noted in cells located dorsally in the brain in the region corresponding to the Kenyon cells of the MB, similar to the previous report (Skoulakis et al., 1993). Nonetheless, further confirmation with a functional PKA activity assay and measurement of constituents of the cAMP pathway or downstream targets (CREB for instance) will be important to conduct in the future to measure treatment efficacy and could be used as biomarkers.

**FIGURE 4:**
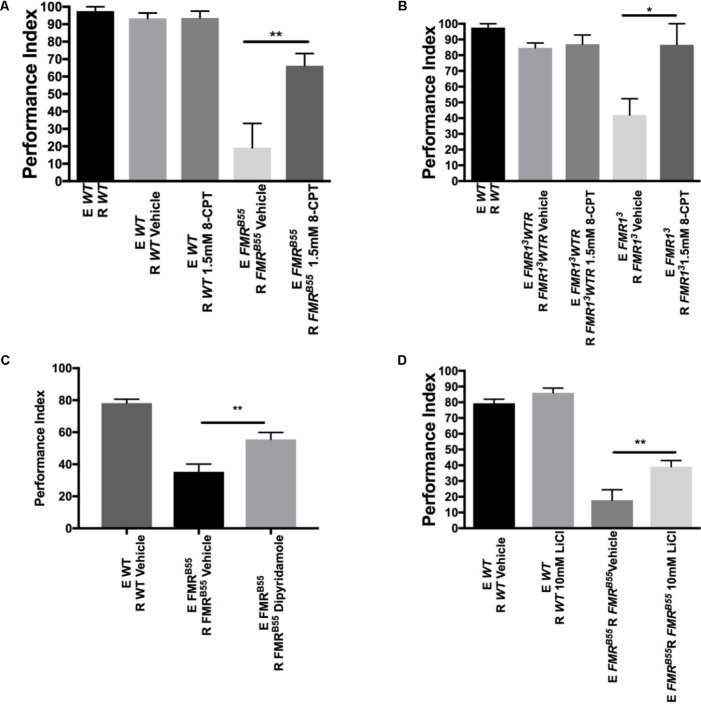
Pharmacological rescue of dSO avoidance with PDE antagonists in *dfmr1* mutant flies. **(A)** *FMR*^B55^ flies treated for 5 days with 1.5 mM 8-CPT exhibited significantly increased avoidance behavior as compared to vehicle (Student’s *t*-test *P* = 0.0073; *N* = 5). 5-day treatment of WT flies with 1.5 mM 8-CPT did not result in any significant difference in avoidance behavior as compared to vehicle (Student’s *t*-test *P* = 0.9688; *N* = 5). **(B)** *FMR1*^3^ flies treated for 5 days with 1.5 mM 8-CPT exhibited significantly increased avoidance behavior as compared to vehicle (Student’s *t*-test *P* = 0.0252; *N* = 6). 5-day treatment of FMR1^3^*WTR* flies with 1.5 mM 8-CPT did not result in any significant difference in avoidance behavior as compared to vehicle (Student’s *t*-test *P* = 0.7334; *N* = 6). **(C)** *FMR*^B55^ flies treated for 1 day with 0.8 mM Dipyridamole exhibited significantly increased avoidance as compared to vehicle (Student’s *t*-test *P* = 0.0064; *N* = 8). **(D)** *FMR*^B55^ flies treated for 5 days with 10 mM LiCl exhibited significantly increased avoidance behavior as compared to vehicle (Student’s *t*-test *P* = 0.0094; *N* = 15). 5-day treatment of WT flies with 10 mM LiCl did not result in any significant difference in avoidance behavior as compared to vehicle (Student’s *t*-test *P* = 0.99; *N* = 15). All graphs depict mean ± SEM. ^∗^*P* < 0.05, ^∗∗^*P* < 0.01.

We wanted to determine whether dSO avoidance behavior could be rescued through pharmacological intervention targeting the cAMP and/or cGMP signaling pathway restricted to the post-natal period as this is closer to potential clinical interventions in individuals with FXS. We first asked whether IBMX, a non-specific cAMP and cGMP PDE inhibitor, could rescue avoidance behavior in FXS flies. IBMX administration for 5 days resulted in a significant increase in avoidance behavior in *FMR*^B55^ and *FMR1*^3^ flies (**Figures 3C,D**). Interestingly, Rolipram, a selective PDE4 (cAMP specific) inhibitor shown to improve olfactory and courtship memory (Choi et al., 2015, 2016), did not lead to significant improvement in avoidance (data not shown). This may suggest that both cAMP and cGMP need to be modulated for rescue of avoidance behavior. We reasoned that other PDEs may be required for dSO rescue in *dfmr1* mutants. Therefore, we used 8-(4 Chlorophenylthio) adenosine 3′,5′-cyclic monophosphate sodium (8-CPT) which is an activator of cAMP-dependent PKA and inhibitor of cGMP dependent PDE. Administration for 5 days of 8-CPT resulted in a significant rescue of dSO avoidance in *FMR*^B55^ and *FMR1*^3^ flies (**Figures 4A,B**). Then, we tested dipyridamole, a United States Food and Drug Administration (FDA) approved drug which increases cAMP levels via both inhibition of PDE-dependent cGMP degradation and adenosine-dependent cAMP synthesis. Dipyrididamole is an inhibitor of PDE 6 which inhibits cGMP and PDE 11 which inhibits both cAMP and cGMP degradation. We observed a significant improvement of *FMR*^B55^ (**Figure 4C**) mutants’ avoidance response after 1 day of treatment. We also tested another drug already FDA approved, Lithium, with effect on cAMP signaling and shown to improve learning and memory in *FMR*^B55^, *FMR1*^3^ flies and FMR1 KO mice (Choi et al., 2015, 2016). We observed significant rescue of avoidance response in *FMR*^B55^ (**Figure 4D**) mutants with Lithium administration after 5 days of treatment (no effect was seen after 24 h treatment – not shown). Together, our pharmacological results strenghten the previous molecular work in FMR1 KO mice showing that FXS may involve both production and degradation of cAMP considering that FMRP binds to mRNAs for PDE regulating cAMP (PDE4B, PDE4DIP, PDE8B), but also cAMP and cGMP (PDE2A) (Darnell et al., 2011).

### cAMP and cGMP Are Linked to Several ID and ASD Genes

**FIGURE 5:**
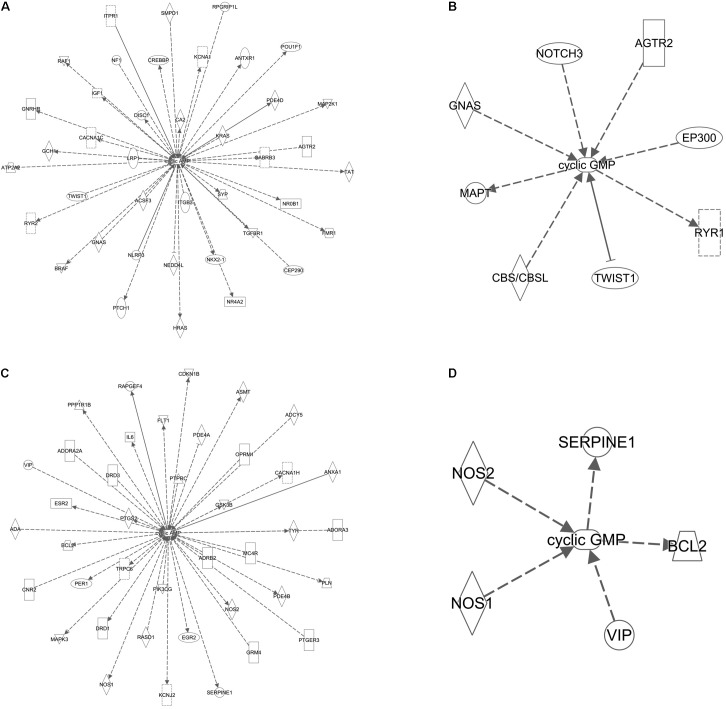
cAMP and cGMP are linked to several ID and ASD genes. **(A)** Gene network of ID genes and cAMP. **(B)** Gene network of ID genes and cGMP. **(C)** Gene network of ASD genes and cAMP. **(D)** Gene network of ASD genes and cGMP. Solid lines indicate direct experimental relationships; dotted lines indicate indirect experimental relationships. Arrows indicate an effect on the target molecule and line arrowheads indicate inhibition.

Based on the recent report of interaction between *FMR1* and several novel ASD candidate genes, we asked if other ID and ASD genes were linked to cAMP/cGMP signaling (Iossifov et al., 2014; Ronemus et al., 2014). This is important as treatment identified for FXS may then be tried in priority with other ID/ASD genes related molecularly. Using an *in silico* gene pathway analysis approach, we identified both ID and ASD genes interacting with cAMP (**Figures 5A,C**) and to a lesser extent cGMP (**Figures 5B,D**).

## Discussion

Our work provides a novel application of dSO avoidance response assay as an endophenotype model to study sensory response behavior in *Drosophila* models of FXS and possibly other ID and autism causes. We show that sensory response required developmental *dfmr1* expression while emission of the sensory cue (dSO) did not. Our results illustrate the importance of *dfmr1* expression in the MB for typical dSO response. This parallels our previous finding showing that *dfmr1* expression in MB was required for learning and memory (Bolduc et al., 2008) although the developmental but also acute expression of FMRP was linked to memory formation.

To our knowledge, this is the first time that dSO defects are rescued pharmacologically in a post-natal setting in *dfmr1* mutants. This is a promising avenue for individuals with FXS suffering of SPD as both Lithium and dipyridamole are FDA approved drugs. As there is pre-clinical evidence showing a conserved deficit of cAMP across species in FXS (Kelley et al., 2007) and recent evidence of improvement of cognitive symptoms in fly and mouse models of FXS (Choi et al., 2015, 2016) with PDE4 inhibitors, our results underline the importance of a symptom specific approach in ID and ASD pharmacological intervention testing. Moreover, PDE-specific inhibitors are currently undergoing clinical trial for behavioral defects in FXS and it may be interesting to assess improvement in SPD. PDEs are well-conserved in flies and include highly conserved critical domains compared to human PDEs (Day et al., 2005). In *Drosophila*, there are seven genes encoding PDEs. The most studied is *dunce* which encodes a PDE4 ortholog and is required for olfactory learning and memory (Kauvar, 1982). More recently, orthologs for PDE1, PDE5 PDE6, PDE8, PDE9, and PDE11 were identified. Our results with IBMX show a strong effect and indicate that multiple signaling cascades may be impacted in FXS. Pharmacologically, IBMX is a complex drug. It inhibits PDE1, PDE2, PDE3, PDE4, PDE5, PDE7, and PDE 11, while PDE8 and PDE9 are insensitive to IBMX. In addition though, apart from its inhibitory effects on PDEs, IBMX has been shown in rat adipocytes to block the inhibitory regulatory protein, G_i_, thereby stimulating AC and increasing intracellular cAMP levels (Parsons et al., 1988). IBMX and other xanthine-derived PDE inhibitors are also well-known adenosine receptor antagonists, consequently increasing cAMP production, which could also be a mode of action as it is “hypoactive” in FXS (Daly et al., 1981; Morgan et al., 1993). Indeed, our results and previous molecular evidence showing that FMRP binds to mRNA of PDEs regulating cGMP suggest that both cAMP and cGMP need to be considered in FXS. As cGMP has been shown to modulate cholinergic and dopaminergic signaling, it is possible that sensory processing requires a tight balance of both cAMP and cGMP (Moody et al., 1981). Maurin et al. (2018) recently showed the importance of PDE2a in FMR1KO mice which has been shown to regulate both cAMP and cGMP. Thus, further molecular dissection studies, for instance using neurons derived from induced pluripotent cells from FXS patients, with more specific PDE inhibitors and AC activators will be required prior to clinical trials.

In addition, our genetic manipulation of FMRP suggests that the defect in avoidance is routed in developmental defects. Importantly though, despite the absence of a clear effect in modulation of FMRP level in adult fly brain on avoidance response, pharmacological treatment of adult *dfmr1* mutants can still improve avoidance performance defects. This implies a potential developmental origin of cognitive dysfunction, but also illustrates that pharmacological treatment should be considered even in absence of acute effect of the target gene on behavior. This raises the possibility that downstream consequences of the absence of *dfmr1* during development, such as dysregulation in epigenetic marks (Korb et al., 2017) and/or structural defects (spine or neuronal network) (Comery et al., 1997; Mansilla et al., 2017) affecting cAMP equilibrium, established during development may be key to the avoidance defects and not the level of FMRP itself. This may be an important consideration when assessing the potential benefit of post-natal treatment in animal models of neurodevelopmental disability.

Finally, treatment targeting cAMP and cGMP may be of benefit to other individuals with neurodevelopmental disorders considering how ID and ASD genes are linked to cAMP-cGMP signaling *in silico*. This raises the need for high-throughput, but clinically relevant systems, to test not only multiple candidate drugs, but several genes.

## Author Contributions

AA co-designed the experiments, performed most of the experiments, analyzed the results, and co-wrote the manuscript. RH performed the *in silico* pathway analyses and assisted in manuscript preparation. SW performed some of the behavioral experiments. CR assisted with the drug preparation and advice on the experimental design. FB co-designed the experiments, analyzed the results, and co-wrote the paper.

## Conflict of Interest Statement

The authors declare that the research was conducted in the absence of any commercial or financial relationships that could be construed as a potential conflict of interest.

## Supplementary Material

FIGURE 1 Spatial requirement and CO_2_ response in *dfmr1* mutants. **(A)** *Feb170-Gal4:UAS-dfmr1RNAi*^1-7^ flies did not exhibit any defect in avoidance response compared to WT flies (Student’s *t*-test *P* = 0.8973; *N* = 10). **(B)** *Feb170-Gal4:UAS-dfmr1RNAi*^1-7^ flies did not exhibit any defect in avoidance when tested against WT dSO (Student’s *t*-test *P* = 0.2119; *N* = 10). **(C)** *FMR*^B55^ (Student’s *t*-test *P* < 0.0001; *N* = 6) and *FMR1*^3^ (Student’s *t*-test *P* = 0.0013; *N* = 6) flies exhibited significantly decreased avoidance to CO_2(g)_ at a concentration of 0.2 mL/min compared to WT flies. **(D)** *FMR*^B55^ (Student’s *t*-test *P* < 0.0001; *N* = 10) and *FMR1*^3^ (Student’s *t*-test *P* = 0.0009; *N* = 13) flies exhibited significantly decreased avoidance to CO_2(g)_ at a concentration of 0.5 mL/min compared to WT flies. All graphs depict mean ± SEM. ^∗^*P* < 0.05, ^∗∗^*P* < 0.01, ^∗∗∗^*P* < 0.001.
