# Supplementary figures and images for: Stress Odorant Sensory Response Dysfunction in Drosophila Fragile X Syndrome Mutants
Source: Front Mol Neurosci. 2018 Aug 8;11:242. doi: 10.3389/fnmol.2018.00242 (PMC6092503; doi:10.3389/fnmol.2018.00242)

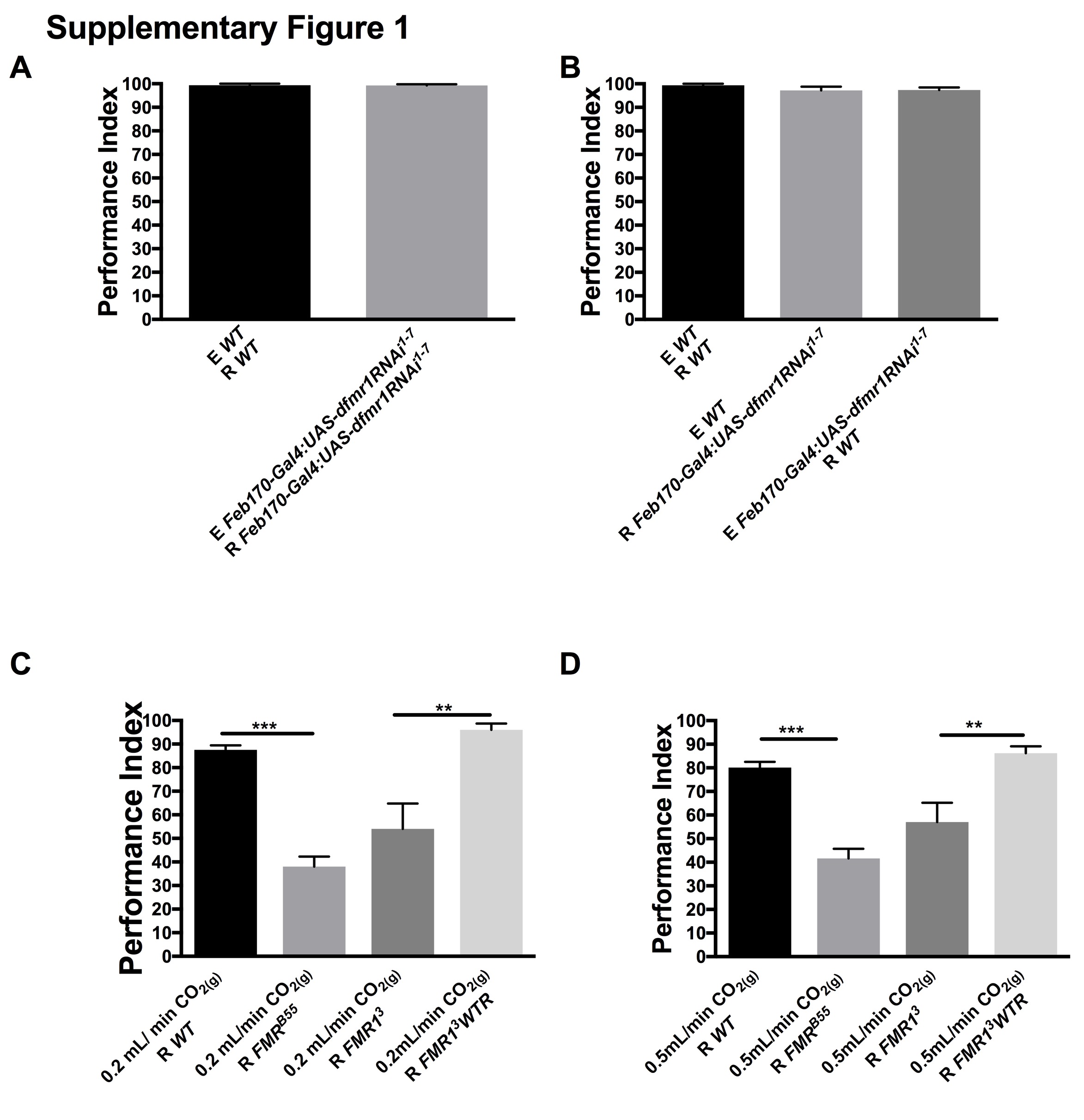

Supplement: FIGURE 1 — Spatial requirement and CO2 response in dfmr1 mutants. (A) Feb170-Gal4:UAS-dfmr1RNAi1-7 flies did not exhibit any defect in avoidance response compared to WT flies (Student’s t-test P = 0.8973; N = 10). (B) Feb170-Gal4:UAS-dfmr1RNAi1-7 flies did not exhibit any defect in avoidance when tested against WT dSO (Student’s t-test P = 0.2119; N = 10). (C) FMRB55 (Student’s t-test P < 0.0001; N = 6) and FMR13 (Student’s t-test P = 0.0013; N = 6) flies exhibited significantly decreased avoidance to CO2(g) at a concentration of 0.2 mL/min compared to WT flies. (D) FMRB55 (Student’s t-test P < 0.0001; N = 10) and FMR13 (Student’s t-test P = 0.0009; N = 13) flies exhibited significantly decreased avoidance to CO2(g) at a concentration of 0.5 mL/min compared to WT flies. All graphs depict mean ± SEM. ∗P < 0.05, ∗∗P < 0.01, ∗∗∗P < 0.001. [file Image_1.jpg]
